# A Case of Abscess of the Brain

**Published:** 1810-05

**Authors:** H. W. Bailey

**Affiliations:** St. Thomas's Hospital


### *A CASE of ABSCESS of the BRAIN.*

ROBERT CLARKE, aged 50, a strong and powerful man, received an injury on the head by a fall, September the 12th; he applied immediately for surgical assistance, and was bled and purged, but the symptoms not being relieved, a blister was applied over his head, which did not afford the least relief. He was then sent to St. Thomas's Hospital, and was taken in, October 26th, 1809, six weeks after he had received the injury. At this time there was a complete paralysis on the left side of the face and of half the tongue, with a numbness in the cheek, and a faultering of articulation; he complained of great pain at the top of his head, on the side which was not paralytic.

Oct. 27, I ordered him a cathartic, and next morning Mr. Birch made an incision upon the part where he described the pain to be seated; after a few days, a considerable discharge took place, and he was much easier; he was kept upon the milk diet of the hospital, and occasionally took aperient medicines. When the wound was nearly healed, he again complained of the return of the pain in his head. Mr. Birch then ordered him pills with gamboge to be taken every night, and directed me to make a crucial incision through the scalp and pericranium. When these incisions suppurated, he again experienced great relief.

By the 3d of December the pain had left him, and the paralytic affection of the cheek and tongue entirely disappeared He seemed lively, and often wished to be discharged from the hospital. In this state he remained nearly two months, viz. till January the 28th. At this time the discharge from the wound was great, and when wiping off the pus I discovered a small hole, which appeared to penetrate both tables of the parietal bone, through which pus was thrown out by jerks; he did not complain of any symptoms which denoted matter upon the dura-mater; the caries extended, and exfoliation was the consequence; after after this, about three days before his death he was seized with cold chills and violent pain in his head; the sight of the left eye became impaired, his pulse low, weak, and very frequent; and he expired Feb. 8, 1810.

*Appearances upon Dissection.*

Having laid bare the cranium, the parietal bone on the right side was found greatly diseased; ulcerations extended through both tables of the bone; this being removed, the dura-mater, corresponding to the diseased portion of bone was found ulcerated; the longitudinal sinus had been much inflamed and thickened, and its cavity was lessened; adhesions were found between the dura-mater covering the right hemisphere of the cerebrum and the falciform process. Upon dissecting off the dura-mater, an abscess was discovered in the posterior lobe of the right hemisphere of the cerebrum, containing about 2 oz. of brown coloured thick matter; this had extended into the right lateral ventricle to the infundibulum, and to the pituitary gland; the thalamus nervorum opticorum was of a dark colour, and likewise the corpus striatum. The brain, on the left side, was in a natural state, except that the plexus choroides was unusually distended with blood. Cerebellum natural.

That so much injury should have communicated within the cranium, and not produce more violent symptoms is worthy of remark.

Had the practice of making an incision through the pericranium, in order to remove the tension of that membrane been adopted directly after the accident, probably the extensive inflammation would have been arrested by the suppuration of the wound, and by the proper evacuations which should also have been directed.

The practice of a free incision on the injured scalp, which Mr. Birch insists upon as being necessary in almost every case, has been attended with such marked success in many violent injuries of the head, that I can venture to say, it should seldom or never be omitted. The hæmorrhage from the contused part is always beneficial; the suppuration of the wound is never followed by exfoliation; and it is only one line of incision, preparatory to what may be necessary, if the symptoms require a further examination of the injury.

I am, &c.

H. W. BAILEY.

*St. Thomas's Hospital,*

*April* 5, 1810.

